# Poliomyelitis Outbreak,Pointe-Noire, Republic of the Congo, September 2010–February 2011

**DOI:** 10.3201/eid1708.110195

**Published:** 2011-08

**Authors:** Arnaud Le Menach, Augusto E. Llosa, Isabelle Mouniaman-Nara, Felix Kouassi, Joseph Ngala, Naomi Boxall, Klaudia Porten, Rebecca F. Grais

**Affiliations:** Author affiliations: Health Protection Agency, London, UK (A. Le Menach, N. Boxall);; European Centre for Disease Control and Prevention, Stockholm, Sweden (A. Le Menach);; Epicentre, Paris, France (A.E. Llosa, K. Porten, R. F. Grais);; Médecins Sans Frontières, Paris (I. Mouniaman-Nara, F. Kouassi);; Médecins Sans Frontières, Brazzaville, Republic of the Congo (I. Mouniaman-Nara, F. Kouassi);; Ministry of Health, Pointe-Noire, Republic of the Congo (J. Ngala)

**Keywords:** viruses, poliomyelitis, epidemiology, infectious disease outbreaks, active immunization, mass vaccination, cross-sectional survey, sanitation, Republic of the Congo, Pointe-Noire, dispatch

## Abstract

On November 4, 2010, the Republic of the Congo declared a poliomyelitis outbreak. A cross-sectional survey in Pointe-Noire showed poor sanitary conditions and low vaccination coverage (55.5%), particularly among young adults. Supplementary vaccination should focus on older age groups in countries with evidence of immunity gaps.

On November 4, 2010, the Ministry of Health of the Republic of the Congo officially declared a poliomyelitis outbreak centered in Pointe-Noire following the laboratory identification of poliovirus type 1 in a patient with acute flaccid paralysis (AFP) (*1**,**2*). A provisional total of 554 AFP case-patients that included a high proportion (68%) of male patients was identified nationally, with paralysis onset from September 20, 2010, through February 27, 2011 (*3*). During this same period, 451 cases (81.4%), which included 184 deaths, were reported in Pointe-Noire. Most cases were found in the young adult population (57.4% in patients 15–24 years of age), which had a higher case-fatality ratio (CFR) of 40.8% compared with the general population of the country (*3*).

In most developing countries, prevention and control of poliomyelitis relies on oral polio vaccine (OPV). Routine vaccination focuses on infants <11 months of age with trivalent OPV, and supplementary immunization activities (SIAs) provide additional opportunities for vaccination for children <5 years of age (*4**,**5*). In response to the epidemic, the Ministry of Health, in collaboration with the World Health Organization, launched 4 rounds of national SIAs that used monovalent type 1 and bivalent types 1 and 3 OPV during November 12–16, 2010, December 3–7, 2010, January 11–15, 2011, and February 22–26, 2011 (*3*). To assess risk factors for infection and estimate vaccination coverage, Epicentre and Médecins Sans Frontières implemented a cross-sectional survey in 1 affected neighborhood of Pointe-Noire.

## The Study

**Figure 1:**
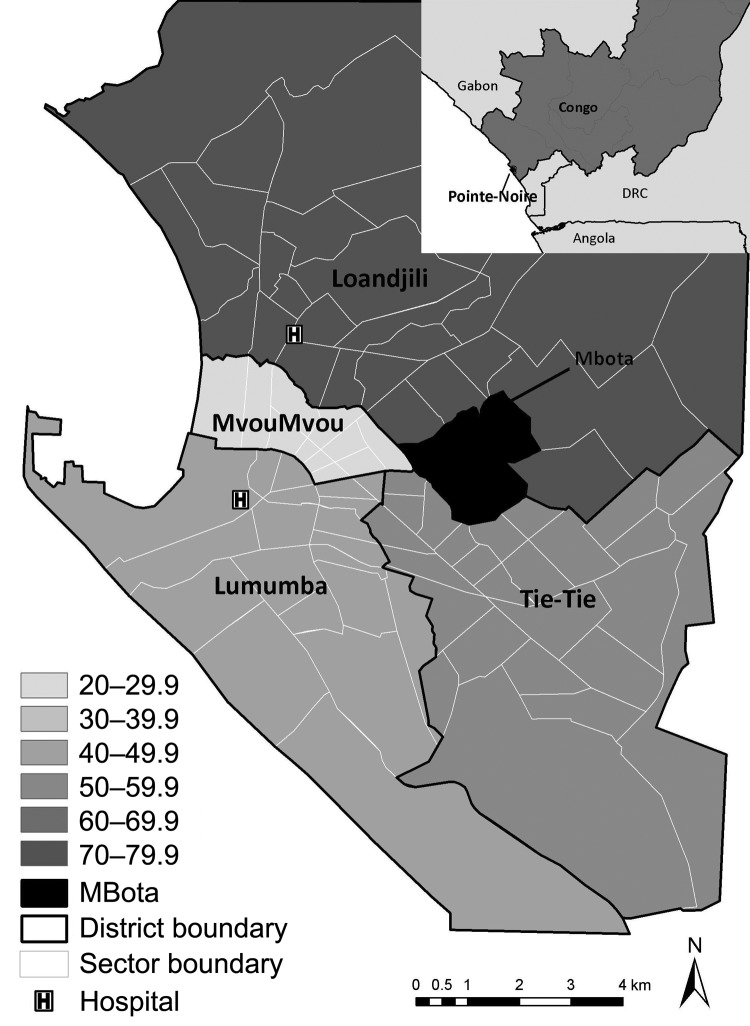
Pointe-Noire, Republic of the Congo, representing the 4 districts, Lumuba, MvouMvou, Tie-Tie, and Loandjili. The gray scale represents the cumulative acute flaccid paralysis (AFP) rate per 100,000 inhabitants September 2010–February 2011. Black area represents the 4 sectors of Mbota where the cross-sectional survey took place. The last census provided by local health authorities occurred in 2007, with a growth rate applied to estimate the 2010 official population size in Pointe-Noire, aggregated by gender and age. We used a 2005 population survey to disaggregate population figures by gender and 5-year age groups and calculate specific acute flaccid paralysis rate. DRC, Democratic Republic of the Congo.

This survey was a single-community rapid assessment of epidemiologic conditions potentially contributing to the outbreak, including vaccination coverage. The survey was done in Mbota, a neighborhood composed of 4 sectors, comprising 9.5% of the city population. This neighborhood was selected from Loandjili district (Figure 1) on the basis of local expert consultation to ensure socioeconomic diversity and representativeness. To estimate vaccination coverage of 80% among children with 5% precision at a 95% confidence level, we aimed to sample at least 246 households. We assumed each household had on average 1 child <5 years of age. We also assumed 5 persons per household on average, which corresponds to ≈1,230 persons overall. Households were selected in each sector of the neighborhood. From a starting point on the border of the sector, interviews focused on every fifth household, following a predefined direction toward the center of the sector, subsequently covering the entire neighborhood. Data were collected through face-to-face interviews December 9–10, 2010, by using a structured questionnaire. The questions included household demographics, water supply, and sanitary conditions. In addition, information for each person in the household was collected about vaccination history (routine and SIAs), any previous illness, and access to health care during the past 3 months. Vaccination coverage was estimated on the basis of reported personal history or documentary evidence of vaccination: a fingernail marked with ink indicating vaccination during SIAs or a vaccination card indicating routine vaccination.

**Figure 2:**
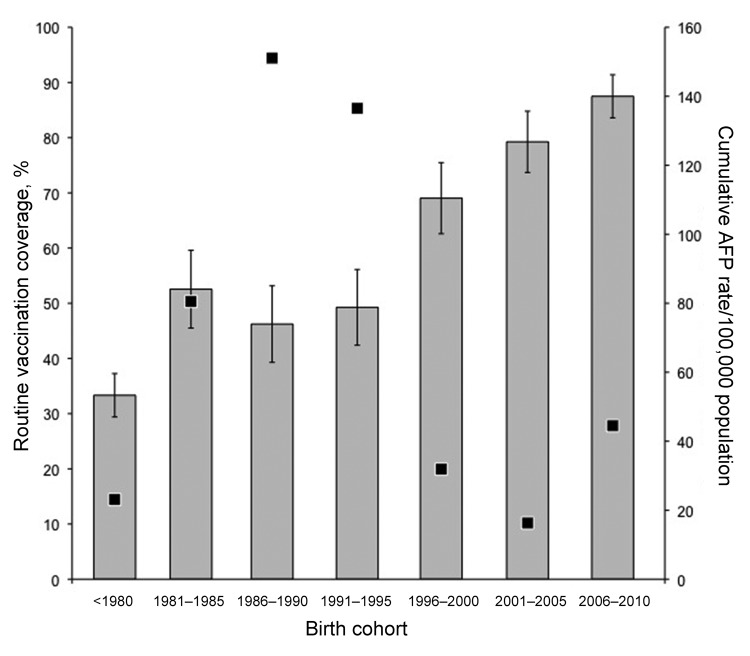
Routine vaccination coverage and acute flaccid paralysis (AFP) rate by birth cohort. Gray bars represents the vaccination coverage according to the survey implemented in Mbota, Pointe-Noire, Republic of the Congo, in December 2010. Error bars represent the 95% confidence interval). Black squares represent AFP rate per 100,000 inhabitants for each of the designated birth cohorts.

Data were eventually collected from 1,849 persons in 317 households of Mbota with all ages and both genders represented. Following the 2 first rounds of SIAs, most surveyed persons (1,812 [98%], 95% confidence interval [CI] 97.3%–98.6%) reported having been vaccinated with monovalent OPV and 1,769 (95.7%, 95% CI 94.7%–96.6%) received 2 doses in November and December 2010. Among vaccinated persons, 627 (33.9%, 95% CI 31.7%–36.1%) had fingernails marked with ink; the rest were absent during the interview or ink had already worn off. There were no significant differences in SIA vaccine coverage by gender or age. Routine vaccination coverage was estimated as 55.5% (95% CI 53.3%–57.8%) among the surveyed persons (Table 1). Among reported vaccinated persons, 21.3% (95% CI 18.8%–23.8%) showed their vaccination card, and among those, 78.7% (95% CI 73.1%–84.4%) were vaccinated >3 times. Routine vaccination coverage did not vary significantly by gender but decreased with age (p <0.0001). For persons younger than 5 years, it was 87.5% (95% CI 83.6%–91.4%) and <52% for anyone >15 years of age. In the most affected age groups, 15–19 and 20–24 years, vaccination coverage was estimated to be respectively 49.3% (95% CI 42.4%–56.1%) and 46.2% (95% CI 39.3%–53.1%) (Figure 2). By comparison, administrative vaccination coverage varied since 1986 between a reported 21% in 1997 and 112% in 2009 (Table 2).

**Table 1:** Persons vaccinated against poliomyelitis (routine vaccination), by age and gender, Mbota, Pointe-Noire, Republic of the Congo, December 2010

Age, y, and gender	No. (%) persons*					Total no. persons
	Vaccinated, card	Vaccinated, recall	Total vaccinated	Nonvaccinated	Unknown	
<5						
M	43 (35.5)	67 (55.4)	110 (90.9)	5 (4.1)	6 (5)	121
F	54 (36.2)	72 (48.3)	126 (84.5)	9 (6)	14 (9.4)	149
Total	97 (35.9)	139 (51.5)	236 (87.4)	14 (5.2)	20 (7.4)	270
5–9						
M	26 (25.5)	53 (52)	79 (77.5)	10 (9.8)	13 (12.8)	102
F	23 (20.9)	66 (60)	89 (80.9)	7 (6.4)	14 (12.7)	110
Total	49 (23.1)	119 (56.1)	168 (79.2)	17 (8)	27 (12.7)	212
10–14						
M	7(7.2)	62 (63.9)	69 (71.1)	9 (9.3)	19 (19.6)	97
F	16 (16)	51 (51)	67 (67)	7 (7)	26 (26)	100
Total	23 (11.7)	113 (57.4)	136 (69.1)	16 (8.1)	45 (22.8)	197
15–19						
M	4 (4.4)	39 (42.4)	43 (46.8)	9 (9.8)	40 (43.5)	92
F	8 (7.3)	49 (45)	57 (52.3)	12 (11)	40 (36.7)	109
Total	12 (6)	88 (43.8)	100 (49.8)	21 (10.4)	80 (39.8)	201
20–24						
M	2 (3)	25 (37.3)	27 (40.3)	7 (10.5)	33 (49.3)	67
F	4 (3.2)	61 (48)	65 (51.2)	14 (*11*)	48 (37.8)	127
Total	6 (3.1)	86 (44.3)	92 (47.4)	21 (10.8)	81 (41.8)	194
25–29						
M	2 (2.3)	37 (43)	39 (45.3)	12 (14)	35 (40.7)	86
F	11 (10.4)	50 (47.2)	61 (57.6)	15 (14.2)	30 (28.3)	106
Total	13 (6.6)	87 (45.3)	100 (51.9)	27 (14.1)	65 (33.9)	192
>29						
M	4 (1.5)	80 (29.2)	84 (30.7)	33 (12)	157 (57.3)	274
F	11 (4.2)	84 (32.3)	95 (36.5)	30 (11.5)	135 (51.9)	260
Total	15 (2.8)	164 (30.7)	179 (33.5)	63 (11.8)	292 (54.7)	534
Totals						
M	88 (10.5)	363 (43.3)	451 (53.8)	85 (10.1)	303 (36.1)	839
F	127 (13.2)	433 (45.1)	560 (58.3)	94 (9.8)	307 (32)	961
Total	215 (11.9)	796 (44.2)	1,011 (56.1)	179 (*10*)	610 (33.9)	1,800

*Card, vaccination documented by card; recall, person remembers received vaccination; total, card + recall.

**Table 2:** Cumulative age-specific AFP rate and vaccination coverage from administrative data and a cross-sectional survey conducted in December 2010, by age group, Mbota, Pointe-Noire, Republic of the Congo*

Age group, y	AFP rate/100,000 persons (95% CI)	Survey vaccination coverage, % (95% CI)	Administrative vaccination coverage, %	5-y average administrative vaccination coverage, %
>29	23.1 (16.9–29.3)	33.3 (29.4–37.3)	†	†
25–29	80.5 (59.2–101.8)	52.6 (45.5–59.6)	†	†
20–24	151.1 (124.3–177.9)	46.2 (39.3–53.2)	41.7–79.0	67.0
15–19	136.5 (112.4–160.6)	49.3 (42.4–56.2)	47–77†	60.5†
10–14	31.9 (20.7–43.2)	69.0 (62.6–75.5)	21–75†	36.7†
5–9	16.3 (8.1–24.6)	79.3 (73.7–84.7)	38–67	53.1
0–4	44.5 (32.2–56.8)	87.5 (83.6–91.4)	81–112	93.9

*AFP, acute flaccid paralysis; CI, confidence interval. †Administrative vaccination coverage provided by health authorities at the country level 1986–2000, at the regional level 2001–2006, and at the city level 2007–2009. Vaccination coverage was partially incomplete for 1992–2000 because of civil conflict. Thus, values for 1994, 1998, and 2000 consisted of interpolated values from years for which data are available.

The number of persons per bedroom, an indicator of crowding, ranged between 1 and 9, and there were >4 persons per bedroom for 12.1% (95% CI 8.4%–15.7%) of all households with no statistical differences among sectors. Tap water either from a neighbor’s house (41.9%, 95% CI 35.9%–47.8%) or within the house (30.7%, 95% CI 25.2%–36.3%) was the primary potable water source recorded. Wells were also common (23.3%, 95% CI 18.3%–28.4%). When the primary source of potable water was unavailable, wells were the most frequently mentioned alternative source (37.8%, 95% CI 32.0%–43.6%). Most households reported use of a latrine shared with neighbors (57.4%, 95% CI 51.8%–63.1%) or within their house (31.4%, 95% CI 26.1%–36.7%). Few surveyed persons (16.1%, 95% CI 14.4%–17.7%) reported illness during the recall period, but if sick, 73.3% (95% CI 67.4%–79.2%) they sought health care, which suggests little underreporting of AFP cases.

## Conclusions

The cross-sectional survey confirmed administrative estimates of low routine vaccination decreasing with age. Specifically, in the surveyed young adult population (15–29 years of age) it varied between a reported 46.2% and 52.6%. According to administrative data, the reported vaccination coverage varies between 41.7% and 79% in young adults between 15 and 24 years of age. Civil conflicts spanning the 1990s have potentially undermined vaccination efforts, especially affecting those currently 10–20 years of age. A further method of protection for a person is past exposure to wildtype strains. To our knowledge, no polio transmission had been reported in Pointe-Noire since the last outbreak in the city in 1969 (*6*). Low vaccination coverage and no previous exposure to wild virus likely led to an accumulation of susceptible persons. Once introduced, the disease spread quickly through this highly susceptible, undervaccinated, and underexposed young adult population, with disease spread facilitated by frequent flooding and poor to medium household sanitation conditions, i.e., crowding sleeping areas, shared latrines between households, and common sources for water consumption.

Poliovirus infections lead to clinical disease more frequently in older age groups (*7*), which partially explains the observed shift of AFP cases toward young adult populations. Moreover, children were better protected with higher vaccination coverage. Death is more frequent when infection occurs in older age groups (*8**,**9*), which explains part of the high observed CFR. Clinical manifestations of poliovirus infections are more common in boys and men than in girls and women (*10*), as observed, and even though males and females share similar vaccination coverage, women in the young adult age groups may have additionally benefitted from exposure to excreted OPV while caring for the young (*11*).

As seen in the Republic of the Congo, or previously in Namibia (*12*), Cape-Verde (*13*), any country with no recent wild-type polio transmission and a similarly low level of vaccination coverage may face similar outbreaks characterized by a large proportion of cases in older age groups and a high CFR (*14*). Avoiding outbreaks will rely on ensuring vaccination of the at-risk population. Routine vaccination activities should be reinforced by SIAs punctually focusing on older age groups (especially among persons 15–35 years of age) when evidence of immunity gaps are documented by serologic surveys or low historical vaccination coverage data. Lessons from the epidemic should benefit health authorities in better prevention and response to further outbreaks on the road to eradication.
